# Feed additives as systems-level modulators of the ruminant host–microbiome interaction: a multi-omics perspective on efficiency, resistance, and reproduction

**DOI:** 10.3389/fmicb.2026.1995937

**Published:** 2026-10-26

**Authors:** Oluwatobi Fijabi, Olanrewaju B. Morenikeji, Shanna Lodge Ivey, Clint Loest, Modoluwamu Idowu, Ryan Ashley, Ibukun M. Ogunade, Godstime Taiwo

**Affiliations:** 1 Department of Animal and Range Sciences, College of Agricultural, Consumer, and Environmental Sciences, New Mexico State University, Las Cruces, NM, United States; 2 Department of Biology, Virginia State University, Petersburg, VA, United States; 3 School of Agriculture, Tennessee Technological University, Cookeville, TN, United States; 4 Division of Animal Science, West Virginia University, Morgantown, WV, United States

**Keywords:** feed additives, feed efficiency, multi-omics, reproductive microbiome, resistome, rumen microbiome, ruminants

## Abstract

Ruminant livestock face converging pressures from climatic variation, antimicrobial agents, and the imperative for sustainable intensification. However, the molecular imprints through which nutritional interventions modulate host–microbiome interactions across physiological contexts remain fragmented across disciplinary themes. Here, we synthesize evidence from metagenomics, metatranscriptomics, metabolomics, and host transcriptomics to propose that feed additives operate as systems-level modulators of a functionally integrated rumen–liver–immune–reproductive axis, rather than acting on isolated metabolic pathways. *Prevotella*, *Butyrivibrio*, *Ruminococcus*, and *Fibrobacter* recurrently emerged across studies to be associated with feed efficiency, immune modulation, resistome dynamics, and reproductive fitness. However, their relationship is context dependent and not causal. Furthermore, hepatic transcriptomic and microRNA (miRNA)-mediated regulatory networks could serve as a molecular bridge connecting ruminal fermentation events to systemic phenotypic outcomes. The ruminant resistome may also represent a potential interface with public health rather than an isolated ecological phenomenon, given the capacity for horizontal gene transfer through mobile genetic elements, although the extent of transmission to humans remains to be quantified. Early-life microbial programming and reproductive tract microbiome composition also represent tractable intervention windows for precision management. This integrated framework could reframe feed additive research from a single pathway to a systems-level perspective, offering a basis for precision ruminant production.

## Graphical abstract

**Figure:**
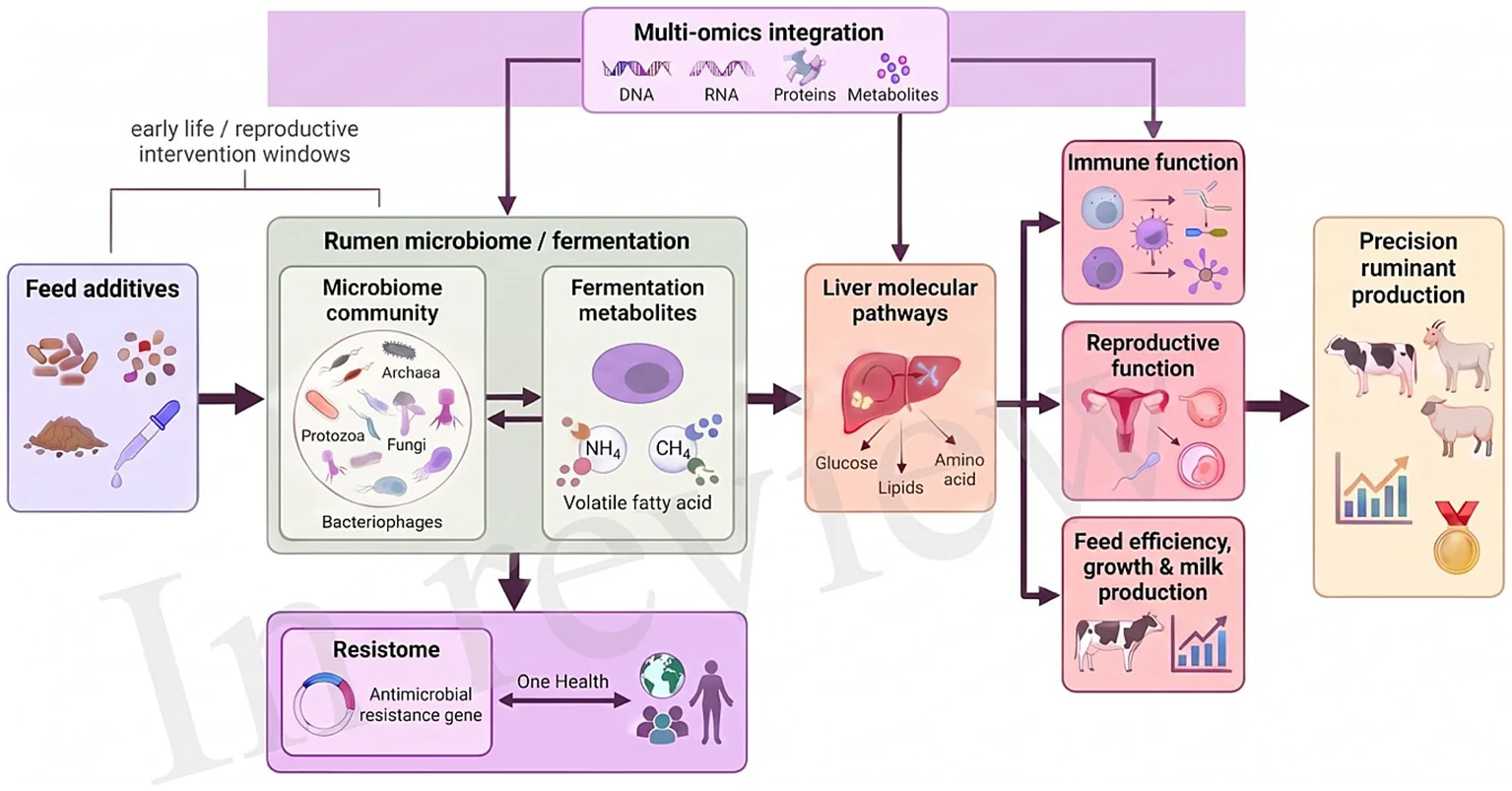
A proposed systems-level framework illustrating the influence of feed additives on early-life and reproductive intervention windows, rumen microbiome composition and fermentation dynamics, resistome profiles, and ruminant metabolism by integrating multi-omics data. This framework can improve growth, milk production, and reproductive efficiencies, immune function, and precision nutritional interventions within a One Health Initiative.

## 1 Introduction

Ruminant livestock contribute approximately 33% of global livestock greenhouse gas emissions and ruminants in developing countries contribute about 70% of this, creating a fundamental tension between food security and environmental sustainability that demands transformative rather than incremental solutions (Dangal et al., 2017; Zhang L. et al., 2022). This challenge is compounded by antimicrobial resistance (AMR), which is identified by the World Health Organization as a top ten global public health threat (World Health Organization, 2023). Global geospatial modeling of AMR prevalence in food animals has identified resistance hotspots across China, India, Brazil, and Central Asia, with 59% tetracycline resistance in *Escherichia coli* isolates and cefotaxime resistance reaching 33% across low- and middle-income countries, underscoring the urgency of evidence-based interventions at the livestock–human interface (Zhao et al., 2024). Reproductive inefficiencies further compound these pressures, resulting in a global loss of genetic gain and billions of dollars annually (Reese et al., 2020). Furthermore, traditional methods for enhancing the efficiency of livestock, like selective breeding for productive and reproductive traits, targeted feeding programs, and prophylactic treatment have achieved considerable success. However, the impacts of these conventional methods are inherently limited because interconnected biological processes are treated as independent variables rather than components of an integrated system.

The ruminant gastrointestinal tract harbors one of the most complex microbial ecosystems on Earth, comprising over 10,000 metagenome-assembled genomes that collectively encode more metabolic functions than the host genome itself (Xie et al., 2021). This community does not merely assist in digestion; it constitutes a metabolic organ that processes dietary substrates into volatile fatty acids (VFAs), microbial proteins, and bioactive metabolites that regulate host gene expression, immune function, and reproductive physiology through what we here term the rumen–liver–immune–reproductive axis (Sasson et al., 2017; Schären et al., 2018). Emerging studies in ruminant nutrition suggest systemic and physiological interactions between rumen microbial dynamics and host responses (Xue et al., 2022; Shao et al., 2023; Li X. et al., 2024; Ajiboye et al., 2025; Liu et al., 2025). However, evidence linking feed additive–induced changes in the rumen microbiota to downstream immune and reproductive outcomes remains limited. Accordingly, the proposed framework depicts putative inter-organ crosstalk and should be regarded as a hypothesis-generating conceptual framework rather than an established mechanistic model.

Feed additives, including ionophores, direct-fed microbials, yeast derivatives, phytogenic compounds, and prebiotic substrates, exert their effects primarily by perturbing this microbial community, yet the mechanistic cascades linking microbial perturbation to host phenotypic outcomes remain poorly resolved at the molecular level.

Traditional approaches studying single genes, metabolites, or microbial taxa in isolation have provided foundational knowledge but cannot capture the emergent properties of the host–microbiome system. Similarly, feedomics and nutrigenomics have emerged as complementary disciplines that examine how dietary variation shapes the omics landscape of both host and microbiome (Sun et al., 2019; Archibald and Joffe, 2021; Liu et al., 2022). Yet these fields have largely developed in parallel, with feed efficiency researchers, immunologists, resistome investigators, and reproductive biologists rarely integrating their multi-omics datasets into unified analytical frameworks.

The emergence of multi-omics platforms is beginning to close this explanatory gap, although significant mechanistic gaps remain. Metabolomics identifies global metabolite changes, metagenomics profiles microbial community composition, metatranscriptomics characterizes microbial functional activity, proteomics quantifies protein abundance, and host transcriptomics captures changes in host gene regulation and expression (Yu Z. et al., 2024). In this review, we synthesized current multi-omics evidence to propose a unified mechanistic framework in which feed additive function as systems-level modulators of the ruminant host–microbiome axis, reprogramming microbial networks, resistome dynamics, developmental trajectories, and reproductive fitness through cross-tissue molecular crosstalk (Figure 1). Despite substantial knowledge in these research areas, available reviews have largely focused on them as independent concepts. To the best of our knowledge, few emerging studies have synthesized these interconnected fields into a single systems biology framework. Accordingly, we organize our synthesis around four interconnected themes: (1) multi-omics insights into feed additive modulation of ruminal fermentation and resource efficiency; (2) the ruminant resistome as a dynamic One Health interface; (3) early-life microbial colonization and developmental programming; and (4) the reproductive microbiome and its integration with nutritional status. We conclude by identifying recurrent microbial and molecular signatures that are notable across these themes, proposing that their occurrence could reflect the fundamental systems architecture of the host–microbiome axis and the nutritional-metabolic fate of ruminants.

**Figure 1:**
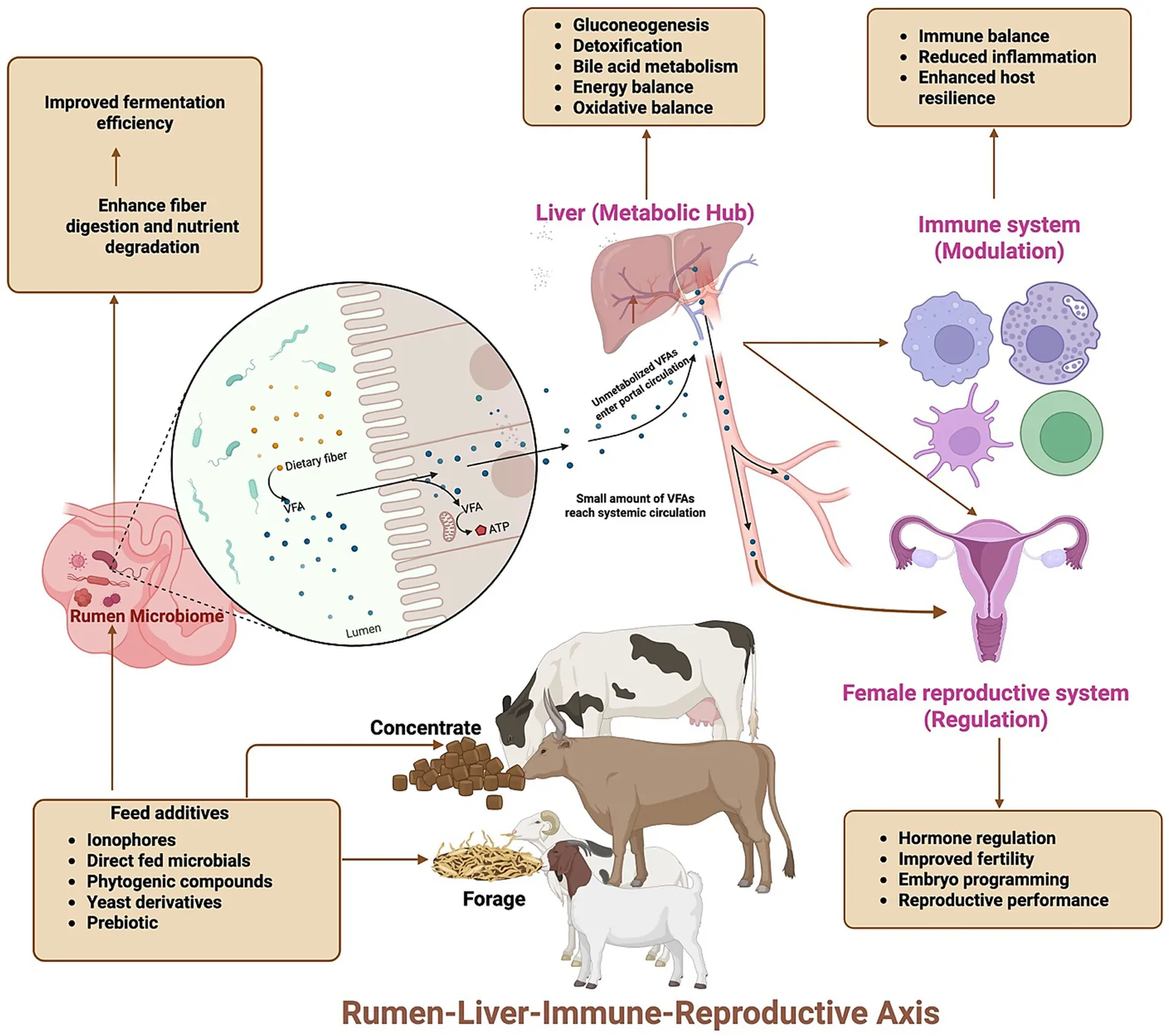
The proposed rumen–liver–immune–reproductive axis framework. Feed additives like ionophores, direct-fed microbial, phytogenic, yeast-derived products, and prebiotics, could first remodel the ruminal microbiome by improving fiber digestion, fermentation, and metabolic efficiency. These ruminal changes alter the production of volatile fatty acids and other microbial-derived signaling molecules, which are absorbed across the rumen epithelium to the liver. Since the liver is a known metabolic hub in ruminant nutrition, it integrates metabolites to regulate gluconeogenesis, bile acid metabolism, detoxification processes, systemic energy balance, and oxidative balance. Through metabolic crosstalk and immune regulation, there is synergistic modulation of immune cell functions to promote immune balance, impede inflammation, and enhance ruminant resilience. Similarly, endocrine and metabolic inputs interact with the reproductive system to shape hormone production, fertility, embryo programming, and reproductive performance. Overall, this framework illustrates the central theme proposed throughout this review: That ruminant nutritional interventions through various feed additives are associated with interconnected host–microbiome molecular networks rather than isolated physiological pathways, eliciting coordinated effects on feed efficiency, immune competence, antimicrobial resistance, developmental programming, and reproductive fitness. Created in BioRender. Fijabi, O. (https://BioRender.com/pnul3um).

### 1.1 Literature search and synthesis approach

We approached the literature search in a structured way to identify studies associated with nutritional interventions with feed additives, the microbiome of ruminants, host physiology, antimicrobial resistance, feed efficiency, developmental programming, reproductive efficiency, and One Health. The literature used here was sourced from online search through PubMed, Web of Science, and Scopus. We combined terminologies like “yeast,” “prebiotics,” “ruminant,” “rumen microbiome,” “probiotics,” “phytogenic,” “multi-omics,” “metagenomics,” “resistome,” “metabolomics,” “transcriptomics,” “antimicrobial resistance,” “early-life microbiome,” “reproduction,” “reproductive microbiome,” and One-Health in a systematic manner. Similarly, relevant literature was sourced through Google searches using those key words to identify other literatures that were not captured by these databases. Studies were selected in alignment with the central theme of this review, with emphasis on recent multi-omics data and their integration in ruminants. We thematically synthesized the studies to expand our knowledge on the influence of feed additive through an integrated system-level host metabolic framework.

## 2 Feed additive reprogram the ruminal microbiome–metabolome–host axis

### 2.1 Fermentation modulation: restructuring the metabolic core

Feed additives exert their primary effects by restructuring the ruminal microbial community toward a metabolically favorable configuration that enhances fiber degradation, VFA production, and nitrogen utilization efficiency. The magnitude of these effects, revealed through integrated 16S rRNA gene sequencing and untargeted metabolomics, extends far beyond simple changes in microbial abundance to encompass fundamental reorganization of interspecies metabolic networks (Wang et al., 2025; Smith et al., 2026; Woody et al., 2026). The efficiency of ruminal fermentation depends on coordinated metabolic coupling among rumen microorganisms that govern carbohydrate fermentation, energy metabolism, nutrient utilization and productivity of ruminants. Ruminal fiber and cellulose digesting species like *Fibrobacter* and *Ruminococcus* produce hydrolytic enzymes capable of metabolizing structural polysaccharides into fermentable substrates (Russell and Rychlik, 2001; Mizrahi et al., 2021). Consequently, fermented substrates produce VFAs and signaling molecules critical to ruminant metabolism and physiological homeostasis. Principally, propionate is a glucogenic precursor in the liver, acetate is associated with lipogenesis and periphery energy metabolism, and butyrate coordinate ruminal epithelia metabolism, nutrient absorption, and ketogenesis (Bergman, 1990). In addition, microbial degradation of nitrogenous and non-nitrogenous compounds produces ammonia and other nitrogenous substrates that can be recycled or assimilated as microbial protein in the presence of ATP (Hackmann and Firkins, 2015).

The redox potential of the rumen is also associated with hydrogen generation and other reducing equivalents that enhance methanogenesis, leading to productive energy loss for ruminant productivity. Acylate and succinate associated propionate-synthesizing pathways reduce the flow of hydrogen for methanogenesis, thereby suppressing the growth of key methanogens, however, their effects are dependent on the availability of substrate and microbial redundancy (Mizrahi et al., 2021). Therefore, feed additives that alter the microbial functional profile could modulate fermentation efficiencies, nitrogen turnover and capture, and methanogenesis. However, the translation potentials of these alterations for enhanced feed efficiencies and productive traits of ruminants remain context dependent especially on the composition of diets, additive type, dosage, duration of supplementation, and host physiology (Hristov et al., 2013; Mizrahi et al., 2021).

Feed additives from different chemical classes can converge on a common mode of action, reshaping the rumen microbial network rather than simply promoting or suppressing individual taxa. Sodium sulfate, an inorganic sulfur source, activates a co-abundance network between *Desulfovibrio* and *Ruminococcaceae* in steers that enhances sulfur amino acid metabolism and fiber degradation through complementary enzymatic cascades (Zhao et al., 2020). The isoflavone phytoestrogen biochanin A, a phytogenic compound, improves nitrogen utilization in dairy goats. Selective enrichment microbial taxa like *Selenomonadaceae* and *Aminobacteriaceae* that express high affinity peptidase and glycoside hydrolase activities. These taxa redirected ruminal nitrogen flux from ammonia toward microbial protein synthesis (Zhang et al., 2026). Microbial feed additive span probiotics (live yeast and bacterial direct fed microbials (DFM)), prebiotics (yeast cell wall mannan oligosaccharides (MOS) and beta glucans), and postbiotics (yeast culture and hydrolyzed yeast), which are widely used to enhance animal performance, health, and production.

Yeast-based additive exemplifies this system-level reprogramming. Integration of 16S rRNA sequencing with LC–MS-based untargeted metabolomics reveal that *Saccharomyces cerevisiae* fermentation products simultaneously increase the relative abundance of fibrolytic taxa (*Fibrobacter*, *Ruminococcus*) and lactate-utilizing bacteria (*Megasphaera*, *Selenomonas*), while depleting starch-fermenting taxa associated with ruminal acidosis (Ogunade et al., 2019; McCoun et al., 2021; Bai et al., 2025). The metabolic consequence of this microbial restructuring was a coordinated shift toward higher propionate:acetate ratios, elevated microbial crude protein synthesis, and reduced methane precursor availability: a configuration that simultaneously improves energy harvest efficiency and reduces enteric greenhouse gas emissions (Bryant et al., 2021). However, the ratio is influenced by the nature of diets, yeast type, and dosage (Baker et al., 2022), and responses may differ across ruminant species. Together these findings show that dietary additive do not merely inhibit or stimulate microbial growth; they reconfigure interspecies metabolic dependencies.

Across these diverse additive classes, a notable similarity emerges: *Prevotella*, *Butyrivibrio*, *Ruminococcus*, and *Fibrobacter* are consistently prevalent across ruminal communities. This prevalence may reflect their roles associated with favorable fermentation profiles in ruminants. Furthermore, this similarity suggests that these taxa may constitute a core functional consortium whose metabolic output including straight and branched-chain VFA serve as the primary signaling metabolites through which ruminal events are communicated to host tissues (Figure 1). Notably, ensemble machine learning analyses indicate that the rumen microbiome accounts for approximately 36% of the variation in residual feed intake, the most robust feed efficiency measure evaluated, suggesting that microbiome-based biomarkers could have practical diagnostic utility for on-farm selection programs (Monteiro et al., 2024).

### 2.2 Hepatic integration: the liver as molecular hub and translator

The liver functions as the primary metabolic hub and translator between ruminal fermentation events and systemic host phenotypes. Hepatic transcriptomics have revealed that feed efficiency-associated ruminal microbiome configurations produce characteristic VFA profiles that activate distinct hepatic gene expression programs through nuclear receptor-mediated signaling (Zhou et al., 2024).

In Nellore beef steers, genome-wide association study (GWAS)-informed liver transcript analysis linked high feed efficiency with hepatic gene clusters enriched in xenobiotic metabolism, Nrf2-mediated oxidative stress response, and cholesterol biosynthesis pathways (Tizioto et al., 2015). Crucially, these hepatic programs were not independent of the ruminal microbiome; the same animals exhibited rumen microbial profiles enriched in propionate-producing taxa whose metabolic outputs serve as substrates for the hepatic gluconeogenic, and lipogenic pathways. This rumen–liver metabolic coupling may suggest that feed efficiency cannot be understood through either microbial profiling or host transcriptomics alone; it emerges from their interaction.

This framework was further investigated through integrated ruminal microbiome, hepatic and whole blood transcriptomic profiling. The results revealed that steers with low residual feed intake (RFI) exhibited upregulation of innate immune genes. Upregulation of genes associated with Toll-like receptor signaling, complement activation, and antimicrobial peptide expression alongside a significantly lower ruminal abundances of *Methanobrevibacter*, *Bacteroidales* BS11 gut group, *Bacteroides*, and *Clostridium sensu stricto 1*. Together, this result suggest that feed efficiency is governed by coordinated host immune–microbiome interactions in tandem with the rumen–liver metabolic axis (Taiwo and Ogunade, 2023; Idowu et al., 2026). Furthermore, Morenikeji et al. (2025) demonstrated that allelic variation in *CYP3A4* and *PLB1* drives feed efficiency-associated immunometabolic divergence, establishing a direct genetic link between host genotype, hepatic metabolism, and microbiome composition. Complementing these host-centric findings, an integrated meta-omics analysis identified six carbohydrate-derived metabolites that distinguished efficient from inefficient rumen microbiomes in dairy cattle. Their result suggest that microbial metabolic output, rather than microbial profile alone, serves as the major driver of feed efficiency divergence and offers candidate metabolic biomarkers for on-farm selection of high-efficient animals (Xue et al., 2022).

Hepatic transcriptomics, microRNA (miRNA)-mediated post-transcriptional regulation, and ruminal microbiome interact as a rumen–liver metabolic system. In this system, microbial fermentation end-products serve as signaling molecules that shape liver gene expression networks through miRNA-dependent regulatory cascades.

In goats supplemented with prebiotic inulin, hepatic glucogenic enzymes such as phosphoenolpyruvate carboxykinase (PEPCK)and glucose-6-phosphatase (G6Pase) were enhanced compared with the unsupplemented group. Metabolomic analysis of the liver equally revealed enrichment in TCA and amino acid metabolites reflecting enhanced availability of substrates for gluconeogenesis (Zhu et al., 2025). Furthermore, Saanen dairy goats supplemented with *Allium mongolicum Regel* flavonoid exhibited changes in their hepatic transcriptomic profile and genes including PTGS1, CSF1R, and ND6. These genes were associated with immunity and mitochondrial metabolism (Xu et al., 2026). In addition, cows supplemented with a mix of menthol, thymol, and eugenol during transition to high grain diet exhibited an enhanced expression of six miRNA transcripts associated with rumen epithelium remodeling (Pacífico et al., 2022). Dairy sheep supplemented with 5% grape pomace showed differential expression of miR-143-3p and miR-382-5p linked to lipid, glucose, and resilience to stress. Downregulation of miR-409-3p and miR-194 associated with PPAR pathways and inflammation were also observed (Guelfi et al., 2026).

Together, these interactions could reframe feed efficiency as a systems-level phenotype governed by coordinated cross-tissue crosstalk rather than a single-pathway trait. This miRNA–mRNA regulatory architecture essentially operates through the RNA-induced silencing complex (RISC), where miRNAs guide AGO2-mediated translational repression or mRNA degradation of target transcripts (Bartel, 2009). The bidirectional characteristics of this regulation reflect that specific miRNAs suppress heat shock proteins (HSP) while heat shock factor 1 (HSF1) driven transcription activates miRNA loci like miR-214, miR-320a, and miR-17-92. This concurrently creates coherent positive and negative feedback mechanisms that could calibrate the magnitude and duration of stress response (Place and Noonan, 2014). This regulatory architecture may be particularly relevant to feed efficiency, as the same miRNAs that modulate the hepatic heat shock response may also target key metabolic genes governing lipid oxidation, gluconeogenesis, and cholesterol biosynthesis.

### 2.3 Immune modulation: the nutrition–immunity interface

The interface between nutrition and immunity represents a critical determinant of ruminant resilience, heat stress, weaning, and dietary transitions. Multi omics evidence now demonstrates that feed additives simultaneously reconfigure the ruminal microbiome and modulate host immune responses, both through VFA and microbially derived peptidoglycans that activate cascades of innate immune signaling via pattern recognition receptors (Akira and Takeda, 2004; Cavallini et al., 2022).

Furthermore, another study demonstrated that yeast culture supplementation mitigated heat stress that induced physiological impairments in growing bulls at multiple levels. At the ruminal level, yeast derived mannans and beta glucans promoted proliferation of fibrolytic and lactate utilizing bacteria, increasing microbial crude protein synthesis and total VFA concentrations. At the systemic level, suppression of malondialdehyde and elevation of glutathione peroxidase activity are suggestive of activation of the Nrf2 mediated antioxidant response. The plasma metabolome revealed a metabolic shift in bile acid, ceramide, and vitamin E metabolism consistent with altered lipid and antioxidant pathways. Collectively, these findings suggest that yeast supplementation confer systemic antioxidant benefits that extend beyond the rumen (Zhang X. et al., 2022). The molecular switches governing this immunological recalibration are increasingly attributed to miRNA-mediated post-transcriptional regulation, with miRNAs acting as integrative nodes that bridge the heat shock response and immune signaling. Furthermore, thermal and inflammatory stress reshape the cellular miRNAome, with downstream consequences for Toll-like receptor signaling amplitude, inflammasome activation, and macrophage polarization (Place and Noonan, 2014). Notably, miR-146a exerts its functions as an anti-inflammatory rheostat by targeting TRAF6 and IRAK1 in the NF-κB cascade (Taganov et al., 2006). Also miR-155 amplifies pro-inflammatory signaling (O'Connell et al., 2007), together creating an immunological set point that feed additive may modulate through their antioxidant and anti-inflammatory effects. Consistent with this, stress-responsive miRNAs including miR-1 (a regulator of HSP70), the miR-17-92 cluster, and miR-214 have been implicated in recalibrating the balance between protein homeostasis and inflammatory activation (Place and Noonan, 2014).

Direct-fed microbial blends and fermentation products show analogous immune-regulatory properties (Figure 1). Whole-blood transcriptomics of beef steers supplemented with multi-species direct-fed microbial revealed upregulation of genes associated with antigen processing and presentation, interferon signaling, and T-cell receptor activation, suggesting that ruminal microbial modulation can reprogram circulating immune cell phenotypes through gut–blood molecular trafficking (Ajiboye et al., 2025). *Astragalus* supplementation in early-weaned lambs simultaneously altered rumen bacterial community structure and elevated serum IgA and IgG concentrations, with correlation network analysis revealing a positive association between *Lachnospiraceae* abundance and levels of immunoglobulin. This crosstalk suggests a mechanistic link between specific ruminal taxa and systemic humoral immune competence in ruminants (Shao et al., 2023).

A synthesizing principle emerges: feed additives tend to simultaneously reconfigure the ruminal microbiome toward enhanced fermentation taxa, elevate VFA and microbial protein output, and activate host anti-inflammatory and antioxidant defense pathways through metabolite-mediated signaling (Figure 1). These are not independent effects but interconnected consequences of perturbing a single integrated system.

## 3 The ruminant resistome: a dynamic one health interface

### 3.1 Architecture and scope of ruminant antimicrobial resistance

The ruminant’s complete repertoire of antimicrobial resistance genes (ARGs), their mobile genetic elements, and the microbial hosts constitute a significant but incompletely characterized component in the global One Health resistance network (Su et al., 2026; Vivarelli et al., 2026). Metagenomic surveillance across diverse cattle populations and production systems has revealed that the bovine gastrointestinal tract harbors ARGs conferring resistance to virtually every class of clinically important antimicrobial, including tetracyclines, macrolides, β-lactams, aminoglycosides, sulfonamides, and last-resort agents such as colistin (de Carvalho et al., 2022; Munk et al., 2024). The clinical urgency of this reservoir was demonstrated by Sun et al. (2020) in a Nature Communications study showing that environmental exposure to livestock farms significantly remodels the human gut microbiota and resistome. Farm workers acquire livestock-associated ARGs not limited to *blaCTX-M* and *fosA3* through shared environmental pathways suggesting that the ruminant resistome is not ecologically contained but can shape human resistance profiles (Figure 2). At the global scale, Li et al. (2025) conducted the most comprehensive analysis of the livestock resistome to date, revealed that mobile, clinically important ARGs hosted by pathogenic bacteria are highly prevalent across all major livestock species, with the convergence of mobility, clinical importance, and pathogen association creates conditions for rapid zoonotic ARG transmission.

**Figure 2:**
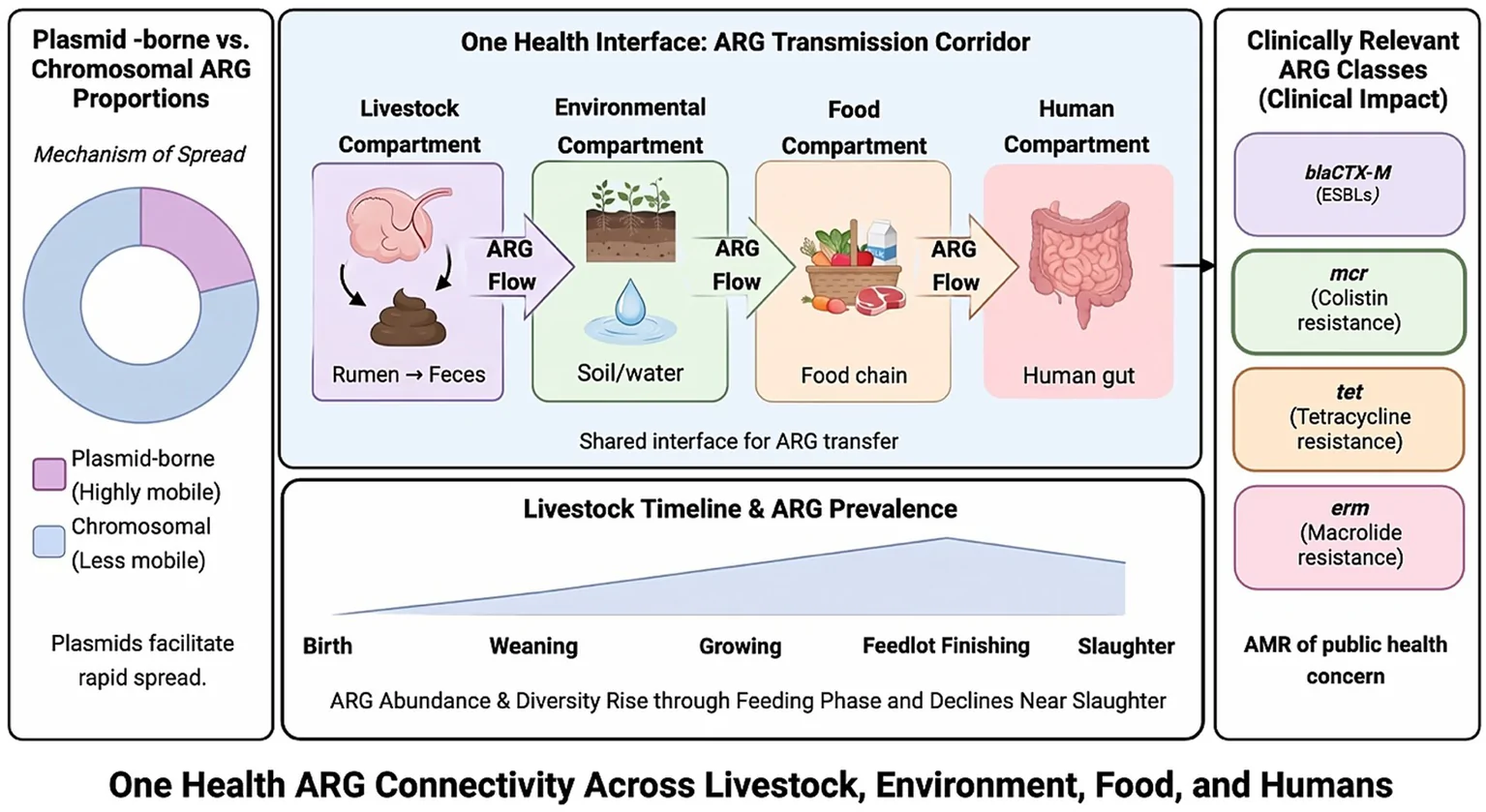
Ruminant resistome dynamics and One Health transmission of antimicrobial resistance genes. This figure illustrates how antimicrobial resistance genes are acquired, their persistence, and means of dissemination within ruminant production systems with implications for One Health. Specifically, the rumen microbiome is the major reservoir of ARGs, encoded with mobile genetic elements, chromosomal DNA with plasmids that enhance horizontal gene transfer among microbial populations (Auffret et al., 2017). As ruminants mature through the production system, ARGs are released into feces and manures which pollute soil and aquatic environments, where they may persist, populate, and recombine. As a result, they can invade and contaminate the food chain from these sources, thereby enhancing their potential for horizontal transmission to the human gut microbiome. Public health important classes of ARGs like *bla*_CTX-M_ (extended-spectrum β-lactam resistance), *mcr* (colistin resistance), *tet* (tetracycline resistance), and *erm* (macrolide resistance) are prevalent across ruminant livestock production systems. The lower panel of the figure shows that the abundance and diversity of ARG abundance appears to increase across the ruminant production system potentially due to increased environmental exposures, biosecurity practices, and antimicrobial selective pressures before reducing or stabilizing after intervention or at slaughter. Created in BioRender. Fijabi, O. (https://BioRender.com/g2uldx1).

The ARG architecture of the ruminant resistome is characterized by consistent core elements alongside substantial geographic and production system variation. An up-to-date comprehensive pan-Brazilian cattle resistome analysis identified *blaCTX-M*, *qnrS*, and *vanG* among the most prevalent ARGs, with plasmid-mediated quinolone resistance genes representing an emergent threat at the livestock–human interface (de Carvalho et al., 2022). In European veal calves, *aph(6)-1d* was identified as the core resistome, with *mef(A)* and *tet(Q)* differentially enriched across production systems (Munk et al., 2024). However, conflicting evidence exists regarding the directionality of resistome changes under production intensification. While Noyes et al. (2016) reported that resistome diversity decreases during feedlot finishing, suggesting convergent selection toward a reduced but clinically relevant ARG pool, Feng et al. (2026) found that grass-fed cattle in western United States harbor distinct fecal resistome profiles compared to grain-fed counterparts, indicating that the relationship between production intensity and resistance may not be linear.

### 3.2 Microbiome–resistome co-evolution

A central insight from resistome research is that ARG profiles are not independent of microbiome composition; the two are deeply intertwined through shared genetic architecture and ecological dynamics. Li et al. (2021) and Cao et al. (2020) demonstrated that specific microbial taxa serve as preferential hosts for some ARG classes, creating stable taxon–ARG associations that persist across geographic and temporal scales. This co-evolution has important implications: feeding strategies, including feed additive, that alter the microbiome can reshape resistome dynamics by selecting for, enriching, or depleting ARG-carrying taxa.

The regulatory landscape is rapidly evolving, with legislative restrictions on prophylactic and growth-promoting antibiotic use expanding globally (Dimuccio et al., 2026). Policy shifts create both an imperative and an opportunity for feed additive-based strategies that can maintain production efficiency while curtailing the selective pressures that drive ARG amplification. Multi-omics surveillance integrating metagenomic ARG detection with metatranscriptomics data will be essential for evaluating whether alternative feed additives genuinely reduce functional resistance or merely redistribute ARGs across different microbial hosts (Figure 3). Vivarelli et al. (2026) recently proposed a novel framework for real-time resistome surveillance that integrates metagenomic monitoring of both livestock and occupationally exposed workers within a unified One Health pipeline, offering a translational model for converting resistome research into actionable public health interventions. Evidence from feed-additive interventions indicates that resistome responses are context-dependent. Thomas et al. (2017) reported that supplementation with monensin and tylosin altered the gastrointestinal microbiota of feedlot cattle, including reductions in selected Gram-positive bacterial taxa, without a detectable association between additive administration and the presence of antimicrobial resistance genes (ARGs). Similarly, Weinroth et al. (2019) found no significant differences in overall fecal microbiome or resistome composition between tylosin-supplemented and unsupplemented cattle. However, geographic region of production was associated with differences in both profiles. Together, these findings suggest that antimicrobial feed additives can alter microbial community composition without necessarily producing detectable changes in the resistome. Conversely, Auffret et al. (2017) reported differences in the abundance and diversity of resistance-associated genes between forage- and concentrate-fed cattle. Such findings should be interpreted within the experimental context rather than generalized to all forage- or concentrate-based production systems.

**Figure 3:**
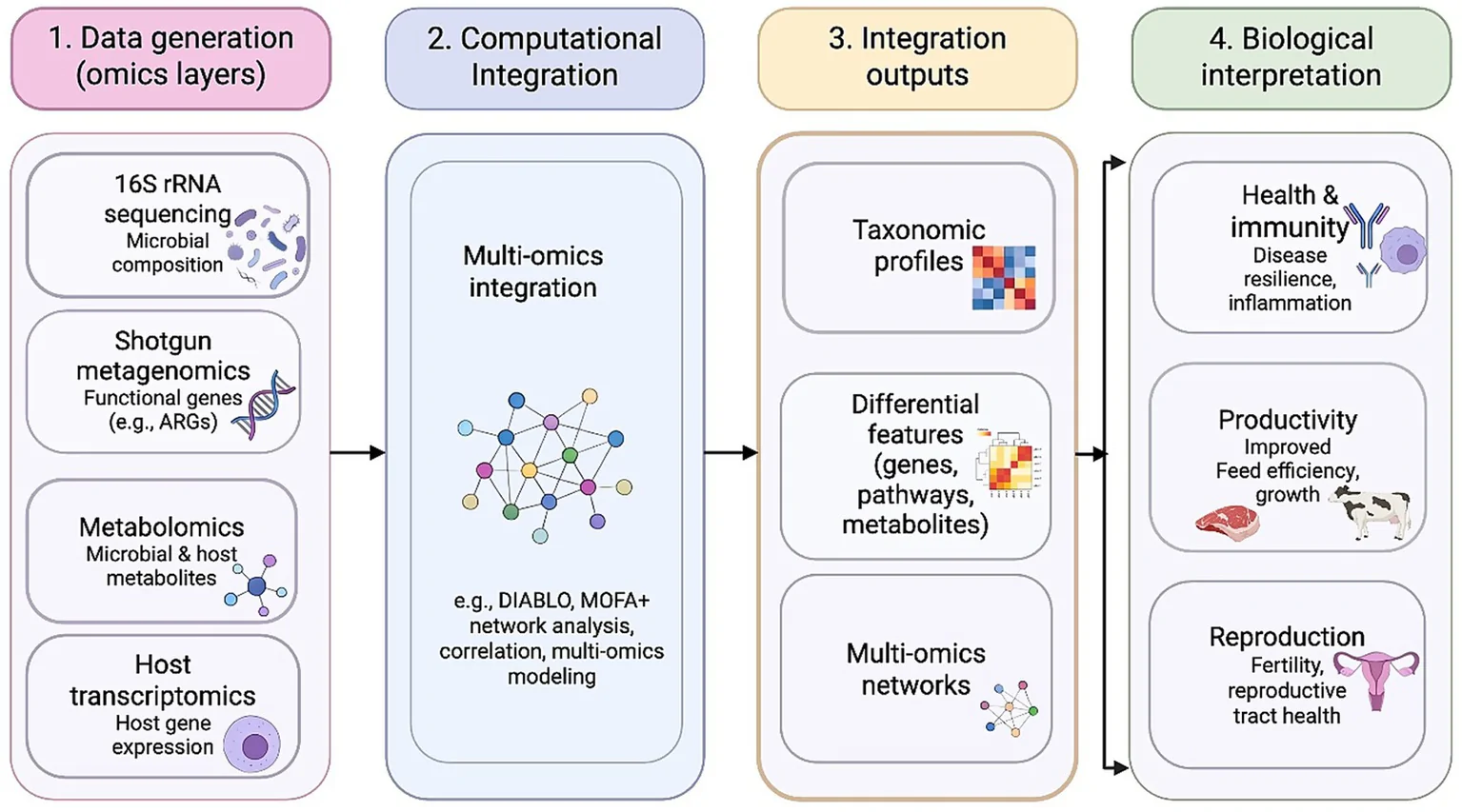
Multi-omics integration framework for systems-level analysis of interactions in ruminants. This framework illustrates how different omics technologies are integrated to profile the molecular mechanisms associated with ruminant health, productivity, and reproduction. Multi-layered datasets like 16S rRNA gene sequencing, shotgun metagenomics, metatranscriptomics, untargeted metabolomics, and host transcriptomics, provide multi-platform complementary information linked to microbial community profile, functional microbial activity, microbial derived metabolites, and host physiological signatures. Big datasets of this nature can be integrated using computational multi-omics approaches, including DIABLO (Data Integration Analysis for Biomarker discovery using Latent cOmponents), MOFA+ (Multi-Omics Factor Analysis), and network-based analytical approaches, to uncover correlations across biological interaction. Outputs associated with these computational approaches are not limited to taxonomic profiles, patterns of functional gene expression, metabolite interaction networks, and host pathway enrichment, enhancing the potential to identify biomarkers and molecular mechanisms linked to ruminant’s feed additives, rumen microbial dynamics, host metabolism, immune modulation, disease resistance, and reproductive efficiency. Created in BioRender. Fijabi, O. (https://BioRender.com/de9fd1c).

Preliminary evidence also suggests that non-antibiotic, microbial-derived feed additives may influence ruminal ARG profiles. Wang et al. (2026) reported significantly lower abundances of tetracycline resistance genes in the rumen of fattening sheep supplemented with *Aspergillus niger* cultures, alongside changes in microbial and metabolite profiles. Nevertheless, findings from both antimicrobial and non-antibiotic feed-additive studies require independent validation across diets, ruminant species, production systems, and management conditions. Changes in ARG abundance alone should not be interpreted as evidence of reduced resistance transmission or lower downstream One Health risk.

### 3.3 Nutritional intervention and resistome dynamics

Feed additives used in nutritional interventions can shape the ruminal and intestinal resistome dynamics of ruminants either through antimicrobial selection or remodeling of the microbial communities. In-feed antibiotics could select for resistant ruminal microbes, while dietary changes can change substrate availability, ruminal environment, microbial dynamics, and ARG-host abundance.

Comparatively, ionophore supplementation on grain fed cattle exhibited distinct ruminal and resistome profile following weaning compared with the grass-fed counterpart. Elevated levels of tetracycline, macrolide, aminoglycoside, β-lactam, and bacitracinβARG were observed in the ionophore-grain-fed group. Notably, a strong correlation between the altered resistome and changes in bacterial profiles was observed. This result may suggest that ruminant production system, nutritional and managerial associated strategies could evolve ecological dynamics that can influence ruminant microbial and resistome kinetics (Kwon et al., 2025). Similarly, Zaheer et al. (2019) established a relationship between beef production system and resistant determinants like metal and biocide resistant genes. This is indicative of the influence of livestock rearing environment in mediating co-selection of resistance dynamics (Zaheer et al., 2019). Within the One-Health context, droppings from livestock could serve as a reservoir of divergent ARGs and determinants, creating a potential path for their transmission to environmental domains (He et al., 2020).

## 4 Early-life microbial programming: the developmental window

### 4.1 Neonatal colonization as ecological succession

The early-life phase represents the most tractable window for nutritional and microbial intervention in ruminants, as the neonatal microbiome is highly malleable before ecological stability is established (Clemmons et al., 2019). Metagenomic evidence indicates that microbial colonization of the neonatal gut initiates before parturition, likely via transplacental microbial transfer, though the extent and functional significance of prenatal colonization remain debated (Funkhouser and Bordenstein, 2013; Guzman et al., 2020). Therefore, transplacental microbial transfer should be regarded as a hypothesis rather than an established path for prenatal colonization. Thus, determining how early-life microbial exposure influences subsequent microbiome development, growth trajectories, and immune competence in ruminants will require longitudinal, contamination-free controlled studies integrating metagenomics with culturomics validation. Evidence consistently suggests that the first weeks of life constitute a critical period during which pioneer microbial communities establish their ecological niche which shapes adult functional profile. A comprehensive synthesis by Lin et al. (2026) proposed that harnessing the early-life gut microbiome represents one of the most tractable strategies for achieving sustainable ruminant production, with interventions during the first three weeks of life producing persistent microbiome alterations that influence lifelong productivity and health outcomes (Figure 4). Notably, Luecke et al. (2023) provided evidence that microbial colonization of newborn calves extends far beyond the gastrointestinal tract to encompass the hoof, liver, lung, nasal cavity, eye, rumen, and vagina, and that prenatal vitamin and mineral supplementation significantly alters these multi-site microbial communities. This suggests that the scope of early-life programming is broader than previously recognized, and that maternal nutritional status shapes neonatal microbiome architecture across virtually every organ and system (Figure 4).

**Figure 4:**
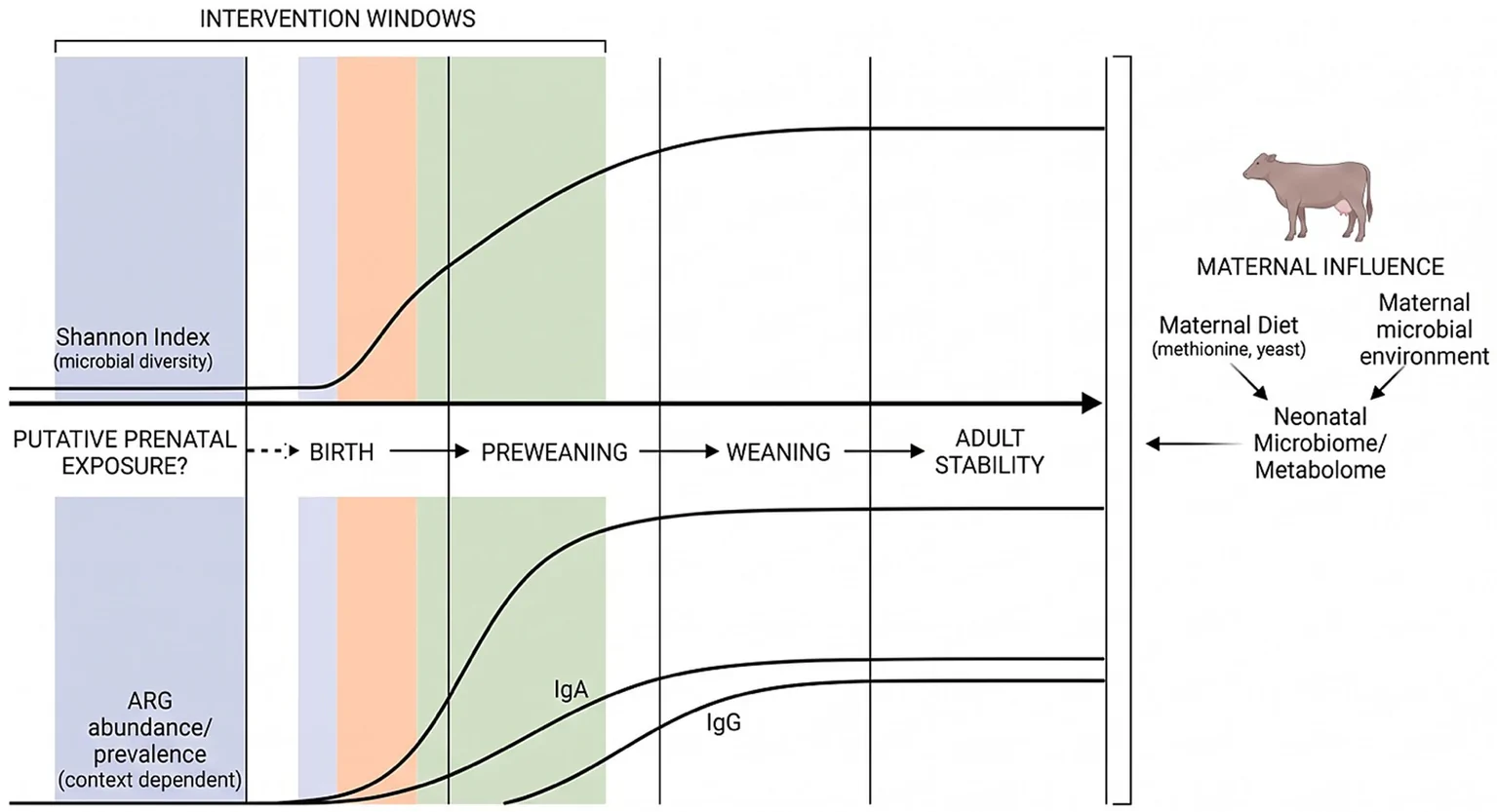
Ruminant neonatal intervention windows, early-life microbial development, and immune maturation. The figure illustrates the proposed temporal dynamics in the prevalence of ruminal microbial communities, ARGs, and the maturation of the immune system from parturition to adulthood. Specifically, transplacental transmission highlighted as putative because current evidence is debatable. Post parturition, maternal influences, including maternal diet (methionine and yeast) and the maternal microbial environment, shape the neonatal microbiome and metabolome, initiating microbial succession that guides growth and developmental trajectories before maturity. Microbial richness (Shannon diversity index) increases from birth through the pre-weaning and weaning stages before stabilizing to an adult microbial community, although the shift is context dependent. Conversely, the prevalence of ARG is at its peak during early life due to environmental exposure, maternal transmission, and antimicrobial selection pressures, before a short decline and thereafter an adult stabilizing profile (Berge et al., 2005; Oh et al., 2020). Furthermore, systemic immune development is evident from colostrum by enhanced immunoglobulin production, especially the secretory IgA associated with intestinal mucosal immunity during early colonization (Stelwagen et al., 2009). Similarly, IgG supports the enhancement of long-term systemic immune protection. The shaded portion of the figure represents a critical intervention window, the growth phase in which nutritional strategies including feed additives, maternal supplementation, and precision microbial interventions could exert the greatest impact on young ruminant’s microbial community assemblies, immune modulation, antimicrobial resistance structure, metabolic programming, and overall feed and reproductive efficiencies. Created in BioRender. Fijabi, O. (https://BioRender.com/2jpkbzm).

The dynamics of this succession follow predictable patterns. Zhang et al. (2019) identified three distinct phases of microbial colonization in goat kids: an initial aerobe-dominated phase, a transitional phase characterized by rapid diversification, and a mature phase approaching adult-like complexity. Critically, perturbation during the early phase can have a pronounced impact on rumen development and ruminant growth trajectories. Consequently, a targeted feed additive strategy during this malleable window could shape and inform neonatal long-term productivity and efficiency.

### 4.2 Maternal nutritional programming of the neonatal microbiome

The influence of maternal nutrition on neonatal microbiome and metabolome represents an example of transgenerational nutritional programming. Periparturient dietary supplementation with rumen-protected methionine altered both the microbial community structure and the metabolomic profile of neonatal calves, with effects detectable from birth through the preweaning period (Elolimy et al., 2019; Elolimy et al., 2020). Calves from methionine-supplemented dams exhibited pathway enrichments in the citric acid cycle, amino acid metabolism, gluconeogenesis, and urea cycle metabolism, which suggests that maternal methionine supplementation may reprogram fetal development and microbiome-associated metabolic functions.

Post-natal interventions during the pre-weaning period are equally consequential for the establishment of microbiome and neonatal health. Prebiotics composed of fructo-oligosaccharides and garlic residue as milk additives selectively promoted growth of *Lactobacillus*, *Bifidobacterium*, and *Faecalibacterium* taxa with established anti-inflammatory and barrier-protective functions (Quijada et al., 2020). Furthermore, Ma et al. (2020) demonstrated that perturbations to the temporal dynamics of neonatal gut microbiota diversity, stability, and composition can predict diarrhea susceptibility with high accuracy.

### 4.3 The neonatal resistome: vertical transmission and early selection

The early life resistome is shaped by the same colonization dynamics that establish the commensal microbiome, with the critical addition that ARGs can be vertically transmitted before any direct antimicrobial exposure (Lin et al., 2026). Haley et al. (2020) similarly showed that pre-weaned calves already harbor a distinct resistome that differs significantly from that of lactating cows in the same facility, suggesting that neonatal ARG acquisition is not simply a mirror of adult exposure but reflects distinct transmission and selection dynamics during the colonization window. This finding has important implications for antimicrobial stewardship, indicating that the neonatal resistome represents an independent intervention target. Summarily, maternal factors, like nutrition, microbial ecology, and metabolic state, may shape early-life microbial programming through interaction between maternal and postnatal pathways. Manipulation of maternal nutrition can influence its microbial and metabolic fate, potentially modulating neonatal microbial composition, through maternal feces, colostrum, and early-life exposures (Elolimy et al., 2019; Luecke et al., 2023). After parturition, microbial changes are a consequence of dietary exposures, antimicrobial use and associated selective pressure, alongside developing immune and metabolic capabilities. These converging processes are potentially the keystone for establishing and maturing resistome profile of neonates (Haley et al., 2020; Ma et al., 2020). Together, this understanding is suggestive of the developmental trajectory connecting maternal nutritional state with young ruminant’s microbial, immunometabolic, and functional resistome profiles. However, there is paucity of reported evidence showing that maternal feed-additive interventions inform a prevalent and persistent offspring’s AMR profiles. Therefore, longitudinal studies which integrate and correlate maternal and neonatal metagenomics, metabolomics, metatranscriptomics, immune phenotyping, and resistome surveillance risk are critical to establish functional effect.

## 5 The reproductive microbiome: connecting nutrition to fertility

### 5.1 Reproductive tract microbiome architecture and fertility

Reproductive performance is the cornerstone of ruminant productivity, driving both long-term economic profitability and genetic improvement (Consentini et al., 2021). The reproductive tract harbors a complex microbiome that extends from the vagina through the cervix and uterus to the oviduct (Figure 5). Vaginal microbial profile varies dynamically across the estrous cycle, pregnancy, and postpartum period (Adnane and Chapwanya, 2022; Poole et al., 2023). Adnane and Chapwanya (2024) characterized these communities and termed them “microbial gatekeepers of fertility”. They revealed that their dynamic shifts across reproductive stages determine whether the uterine environment support or antagonize embryo implantation and development, with culture-independent metagenomics revealing far greater microbial complexity than previously appreciated through conventional and culturomics approaches.

**Figure 5:**
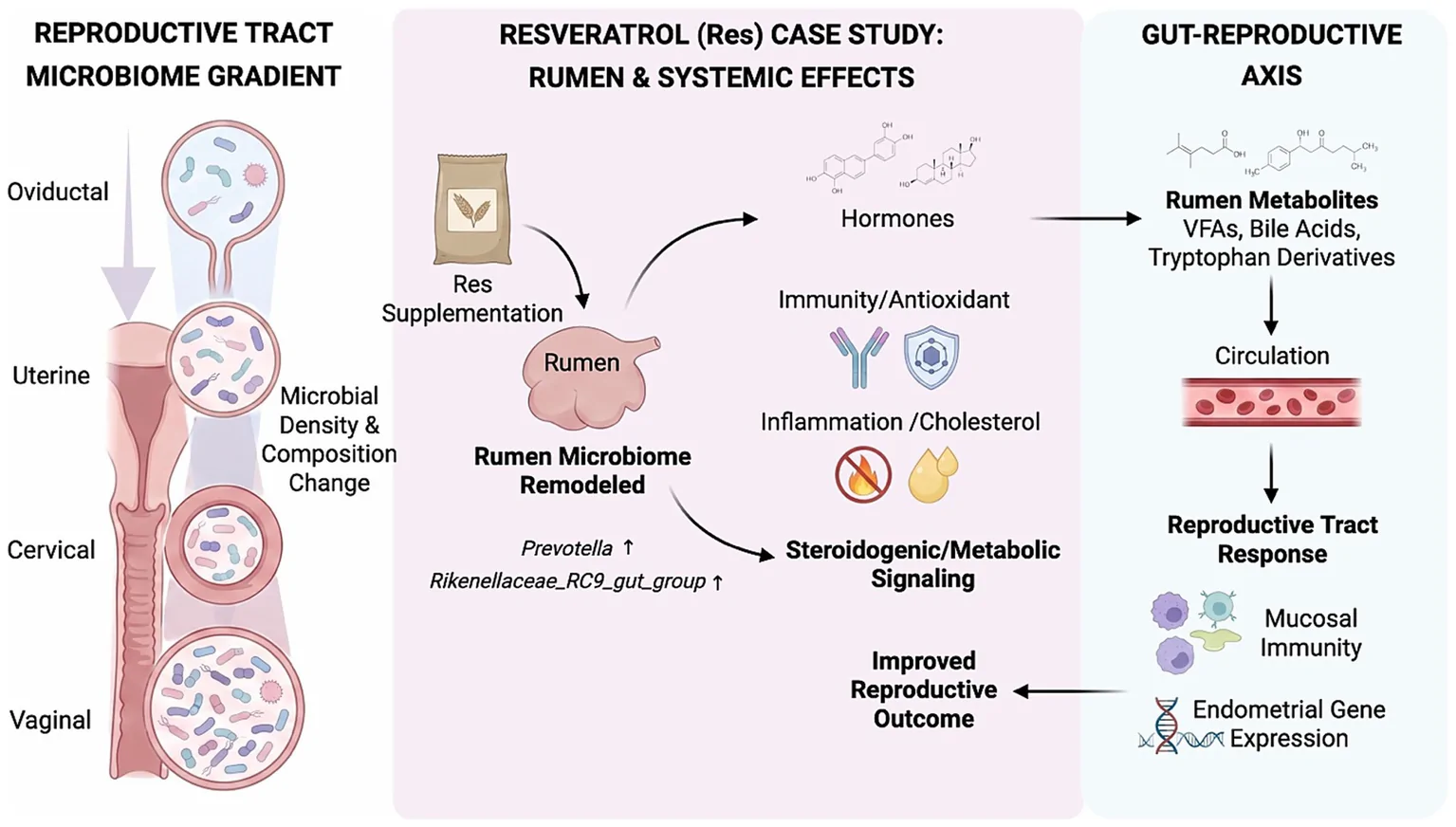
The reproductive tract microbiome, dietary resveratrol supplementation, and the gut–reproductive axis in small ruminants. This figure illustrates the dynamic changes in microbial communities along the small ruminant female reproductive tract, and how dietary interventions and systemic metabolic signaling influence reproductive capacity. The microbial gradient across the vaginal, cervical, uterine, and oviductal compartments is presented on the left panel, indicating lower microbial density from the lower to the upper reproductive tract. These compartment-dependent microbial profiles shape reproductive tract immunity and fertility. The central panel illustrates the ruminal influence of resveratrol; a phytogenic feed additive, enriching beneficial microbial taxa, like *Prevotella* and *Rikenellaceae_RC9_gut_group*. These microbial shifts enhanced antioxidant capacity and immunity, reduced inflammation and cholesterol metabolism, while modulating steroidogenic and metabolic signaling pathways with an overall improvement in reproductive performance (Li X. et al., 2024). The right panel illustrates the gut–reproductive axis, in which rumen-derived metabolites, like the volatile fatty acids (VFAs), bile acids, tryptophan metabolites, and microbial signaling molecules, proceed to the systemic circulation with direct capacity to exert an influence on the dynamics of the reproductive tract. These circulating metabolites shape mucosal immune events, stimulate the patterns of endometrial gene expressions, thereby contributing to enhanced fertility and reproductive efficiencies. Created in BioRender. Fijabi, O. (https://BioRender.com/pvtpgy0).

Metagenomic analyses have also transformed our understanding of reproductive tract infections. Amplicon sequencing of metritis-affected cows consistently revealed reduced microbial diversity relative to healthy controls, with enrichment of pathogenic taxa including *Fusobacterium necrophorum*, *Bacteroides*, and *Porphyromonas*. This is concurrently accompanied with the depletion of commensal *Lactobacillus* species (Jeon et al., 2015; Sicsic et al., 2018). Furthermore, the endometrium in diseased animals shows elevated expression of inflammatory proteins including IL-1β, IL-6, CXCL8, and CCL5, alongside dysregulation of oxytocin receptors and prostaglandin biosynthesis pathways. This profile could represent a transcriptomic signature that directly links microbial dysbiosis to impaired reproductive function at the molecular level (Fagundes et al., 2019). However, reproductive efficiency and microbiome associated studies should be interpreted with caution due to several confounders. The vaginal microbial profiles are influenced by nutritional condition, hormonal profile, stress, and management practices linked to the host (Gupta et al., 2024). Notably, a meta-analysis of more than 56,000 diagnostic records from 159 studies estimated that approximately 48% (47.9%) of beef cows subjected to a single insemination were not pregnant by the end of the first month of gestation. This reflects a combination of fertilization failure and early embryonic loss rather than pregnancy loss among confirmed-pregnant animals alone (Reese et al., 2020). However, there is evidence that suggest a relationship between vaginal microbial profile and fertility outcomes (Luecke et al., 2022).

Single-cell transcriptomics has added high resolution to conceptus–endometrium interactions. Jia et al. (2023) characterized conceptus elongation and implantation dynamics at the single-cell level in sheep, identifying cell-type-specific gene expression programs that govern the maternal–fetal interaction during the critical peri-implantation window. These findings suggest that the uterine microbiome may influence fertility not merely through inflammatory pathology but through modulation of the molecular signals governing implantation. Furthermore, higher pregnancy outcomes, with reduced time to conception, and downregulated expression of inflammatory chemokines (IL1B, IL8, CXCL) resulted from the intrauterine administration of *Lactobacillus buchneri* in dairy cows with subclinical endometritis (Peter et al., 2018). Genís et al. (2018) similarly reported that intravaginal administration of lactic acid bacteria altered the expression of inflammatory neutrophil genes and reduced the prevalence of postpartum metritis. Conversely, intravaginal probiotic administration did not influence conception rate in dairy cows (Madureira et al., 2023). These results suggest the contextual reproductive tract responses of ruminant species to microbial interventions. The male contribution to reproductive tract microbial ecology has been increasingly recognized. Luecke et al. (2022) provided an important framework demonstrating that the seminal and vagino-uterine microbiomes interact dynamically during mating and artificial insemination, with semen-introduced microbes either complementing or disrupting the resident uterine microbiome and thereby influencing fertilization success.

### 5.2 The gut–reproductive axis: feed additives bridge nutrition and fertility

Resveratrol supplementation in ewes across the estrous cycle, pregnancy, and lactation period provides an important example of the gut–reproductive axis (Li X. et al., 2024; Figure 5). Dietary resveratrol simultaneously altered the ruminal microbiome, enhanced systemic antioxidant capacity, and improved reproductive tract immune defense. Similarly, serum analyses revealed significant correlations between ruminal microbial genera and key hormonal markers. *Prevotella*, *Prevotellaceae* UCG-001, and *Ruminococcus* were positively correlated with estradiol and progesterone concentrations while showing negative correlation with inflammatory markers, suggesting a microbiome–endocrine–reproductive signaling pathway (Li X. et al., 2024). Other nutritional interventions reinforce this concept across species. In ewes, L-citrulline and isochlorogenic acid supplementation sustained across pregnancy and lactation each reshaped the rumen microbiota and its metabolites alongside shifts in reproductive hormones, immune markers, and estrus or conception outcomes (Li S. et al., 2024; Sha et al., 2024; Liu et al., 2025). In cattle, feed additives extended these effects to the reproductive tract itself, where rumen-protected choline, *Bacillus,* and essential oil supplement altered the vaginal, endometrial, and rectal microbiomes, with choline lowering the metritis pathogen *Fusobacterium* (Marques et al., 2024; Yildirim et al., 2025). Comparable effects mediated through microbiome remodeling reach male fertility, where melatonin reshaped the gut microbiota to alleviate heat stress spermatogenic dysfunction in dairy goats (Guo et al., 2024).

Collectively, these findings are significant because they demonstrate that feed additives and physiological transitions can inform the microbiome and metabolic outputs, with a consequent influence on ruminant immune response, endocrine signaling, microbial ecology of the reproductive tract, and neonate development. The emerging evidence highlighted above provides support for the rumen-endocrine-reproductive synergy though not through a linear path. Manipulation of the reproductive microenvironment through feed additive remains an emerging area with substantial potential. However, it requires the proposed integrated multi-omics approaches with longitudinal studies outlined throughout this review to understand the molecular imprints linking dietary intervention to fertility outcomes.

## 6 Synthesis: convergent signatures and an integrative framework

### 6.1 The recurring microbial and molecular nodes

A consistent pattern emerges when the evidence across feed efficiency, immunity, resistome dynamics, and reproductive biology is examined through a systems lens rather than disciplinary silos. Across the body of work synthesized here, *Prevotella*, *Butyrivibrio*, *Ruminococcus*, and *Fibrobacter* were associated with nutritional interventions and fermentation phenotypes. Their recurrent relationship with productive and fermentative traits suggests that these taxa could be putative microbial markers, indicative of the nutritional and fermentative fate of ruminants. Thus, they could constitute a functionally integrated fermentation consortium whose metabolic outputs especially the straight and branched chain fatty acids function as signaling molecules. These molecules may activate receptors on metabolic organs, stimulate inflammatory cascades, and shape ARGs dynamics with the potential of modulating reproductive tract competence and systemic metabolic trafficking (Figure 1).

Similarly, the convergence of the microbial taxa may not be coincidental. It may reflect the fundamental architectural principle that the ruminant host–microbiome axis operates as a single integrated system in which perturbation at any node propagates through metabolic, immune, and endocrine signaling networks to influence distal tissues and functions. Notably, the immunity–metabolism synergy identified at the genomic level through GWAS and hepatic transcriptomics may mirror the simultaneous immune and metabolic effects of feed additives observed at the transcriptomic and metabolomic levels. This could suggest that host genetic variation and microbial community structure may co-evolve to optimize similar integrated physiological networks. The One Health dimension of these convergences may extend beyond the ruminant host. Exposure of livestock to the environment can remodel the human gut microbiome and resistome (Sun et al., 2020), this can position the ruminant host–microbiome axis not as an isolated agricultural system but as a node in the global ecology of antimicrobial resistance. In addition, the miRNA regulatory layer adds an important dimension to this system-level convergence. There is limited evidence revealing a putative synergy between intercellular and inter-organ interactions. Dairy cattle with dietary modification exhibited altered circulating serum extracellular vesicle (EV) mediating the expression of miRNAs associated with hormone signaling, immune responses, and metabolic pathways. This provides an insight into the potential mechanism underlying the processes through which dietary and microbiome perturbation of ruminant can be perpetuated beyond the rumen and gastrointestinal tract (Quan et al., 2019). Similarly, Huang et al. (2026) also revealed that dairy cow under heat stress manifest symptoms of rumen dysbiosis, altered plasma circulating metabolites and EV-derived miRNAs in milk. A rumen-mammary communication axis was proposed due to miRNA targeting immune and stress response, hormonal regulation and mammary gland development and biogenesis. Data from an in-vitro sheep study revealed that immune function is associated with EV-miRNA signaling. The miRNA cargo of EV released from the peripheral blood mononuclear cells (PBMC) was altered by inflammatory signals. Although there was no evidence of a rumen associated EV-inter-organ communication, it however showed that immune cells of ruminant have potentials of producing EV-miRNA cargo capable of eliciting systemic response (Ciliberti et al., 2023). This miRNA interaction could enable inter-organ communication between the gut, liver, and immune compartments, with exosomal miR-155 and miR-146a cargo from intestinal epithelial cells proposed to reprogram the polarization state of hepatic macrophages and circulating monocytes (O'Connell et al., 2007; Valadi et al., 2007). This mechanism provides a molecular basis for how ruminal microbial perturbations translate into systemic immune phenotypic changes. Specifically, absorbed ruminal metabolic outputs that reach the liver could act in concert with miRNA-loaded extracellular vesicles that propagate regulatory signals across tissue boundaries. Thus, the EV-miRNA axis may represent a mechanistic bridge connecting the themes of this review. Similarly, circulating exosomal miRNA profiles may serve as a tractable biomarker platform and a non-invasive indicator of microbiome–host immune status.

### 6.2 Multi-omics integration: opportunities and current limitations

The studies reviewed demonstrate that single-omics approaches, while valuable, systematically underestimate the complexity of host–microbiome interactions. The consistent finding that microbiome and host datasets reveal complementary rather than redundant information supports the value of multi-omics integration for ruminant systems, although, as outlined below, several analytical and biological constraints currently limit its routine application and it should not yet be regarded as a default methodology. Methods such as DIABLO (Data Integration Analysis for Biomarker discovery using Latent cOmponents), MOFA+ (Multi-Omics Factor Analysis), and network-based approaches offer the statistical frameworks necessary for this integration, illustrated in Figure 3. However, their application in ruminant science remains limited compared to human microbiome research. Nevertheless, DIABLO can identify coordinated multi-omics signatures associated with functional phenotypes. MOFA+ can identify conserved and intrinsic factors across big data (Singh et al., 2019; Argelaguet et al., 2020).

Furthermore, because of the dimensional, compositional, batch effects, and sparsity characteristics associated with omics datasets, it makes normalization and biological interpretation difficult (Thomas et al., 2017; Santiago-Rodriguez and Hollister, 2021; Yu Y. et al., 2024). These flaws are critical for livestock experiments where diet, physiological stage, breeds, production system, sampling and analytical framework could present biological and technical artefacts. Therefore, correlations observed from either of these integrative approaches could reflect a shared biological response to dietary, breed, and other environmental factors. Thus, future ruminant multi-omics studies should as a matter of priority carry out controlled longitudinal experiments, alongside unified processing, large replication, batch effect control and validations through rigorous statistical analyses to remove noise that could represent artefacts.

### 6.3 From molecular markers to precision management

The breadth of molecular and microbial markers identified through omics approaches create a pathway toward precision ruminant management that integrates microbiome monitoring, metabolic profiling, and host genomic data into actionable decision tools (Figure 6). By synthesizing these themes within a unified framework, ruminant scientists could identify molecular and immunological imprints, and microbial markers that can inform precise feeding strategies, genomic selection programs, and reproductive management practices for a sustainable ruminant production system.

**Figure 6:**
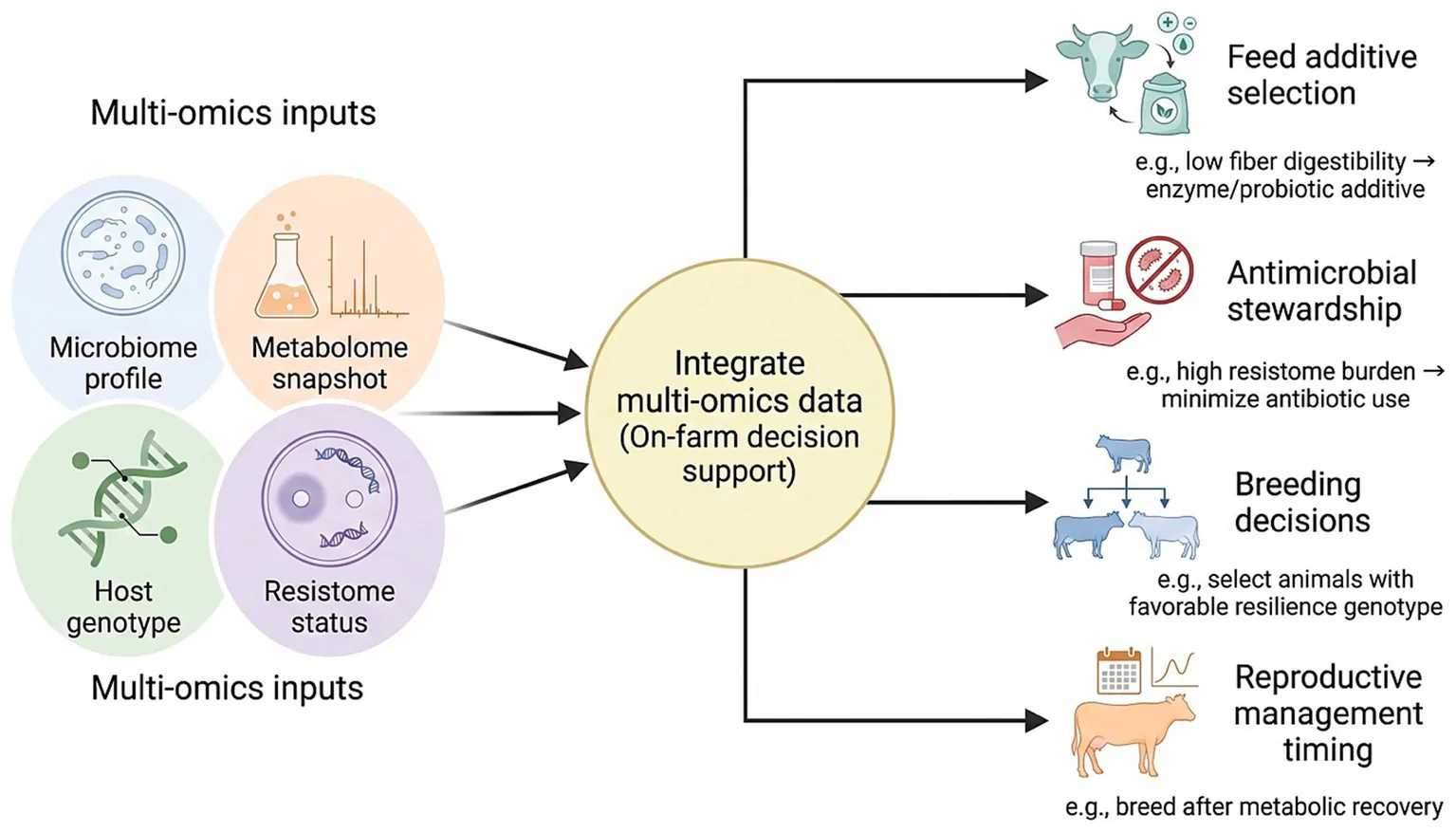
Proposed multi-omics ruminant management framework for on-farm precision nutrition support. This ruminant management framework illustrates how big data generated from multilayered omics platforms can be integrated and translated into informed strategies for improved animal health, productivity, and sustainability. Complementary big data from ruminal microbiome profile, metabolome biomarkers, host genetic variation, and resistome status can be integrated through computational platforms to inform management strategies and recommendations. The resulting systems-level output can inform four major decision pathways. 1. Selection of feed additive: Dietary supplementation can be optimized through insight from the microbial profile and metabolic status associated with improved fiber degradation, nutrient utilization, and feed efficiency. 2. Antimicrobial stewardship: Consistent resistome longitudinal profiling empowers antimicrobial stewardship. Such stewardship encourages and enhances producers to cut down on antimicrobial use, identify the risks associated with resistance, and implement targeted interventions to reduce ARG transmission. 3. Breeding decisions: Insight from breeding studies stems from associating host genomic data with microbial and metabolic biomarkers to select animals with superior feed efficiency, disease resilience, and reproductive potential. 4. Reproductive management timing: Timing decisions are informed by integrating metabolic and physiological markers to predict the best breeding window for artificial insemination and synchronization, thereby improving conception rates and reproductive performance. Created in BioRender. Fijabi, O. (https://BioRender.com/9grf02y).

## 7 Conclusion and future perspectives

Evidence synthesized across the reviewed works support the view that feed additives can operate through interconnected domains involving the rumen microbiome, systemic metabolism, immune responses, and reproductive physiology rather than through isolated biological processes. Collectively, our synthesis suggests an association between the rumen microbial profile and their functional roles with feed efficiency, immune responses, resistome, developmental programming, and reproductive physiology. However, large variation exists across ruminant species, diet and additive type, level and duration of supplementation, production systems, and analytical pipelines. The strength and direction of these relationships remain largely inconsistent due to these confounders. Therefore, the proposed rumen-liver-immune-reproductive framework is a conceptual model that integrates existing and emerging evidence rather than an established mechanistic pathway.

Several limitations from current evidence were identified. The body of knowledge that currently governs the understanding of ruminant nutrition is largely heterogenous and cross-sectional in nature. Such designs obscure causal inferences and direct comparative analyses across ruminant production systems. Therefore, longitudinal design, unified multi-omics pipelines and controlled experiment with validated mechanistic designs are warranted. Specifically, harmonized framework for integrating metagenomics, metabolomics, ruminant transcriptomics and antimicrobial resistance profile could improve resolution of the correlations that are intrinsic among ruminant breeds and genetics, microbial variations, molecular and production phenotypes. Furthermore, controlled, multi-site longitudinal studies are warranted to investigate the effects of nutritional interventions on ruminal and reproductive tract microbiomes and their associations with reproductive outcomes, including estrous synchronization responses, conception rates, pregnancy maintenance, parturition success, and neonatal health. Such studies should also examine maternal–neonatal microbial transmission, including potential contributions of placental and reproductive tract microbial exposure, while accounting for the ongoing debate surrounding the existence of a resident placental microbiome. Therefore, an approach of this nature should account for biological and environmental confounders like breeds and diverse population, geographical and production systems, diets, and managerial strategies, which can translate to practical animal nutrition applications.

Finally, our proposed system-level framework could be a springboard toward One Health applications. Instead of implying causality, the framework has potential of linking ruminant nutritional modulation of the rumen microbiome with resistome alterations, environmental and food chain transmission, with direct implications on human health. Thus, our capacity to translate these microbial and molecular signatures into practical precision ruminant feed formulation, antimicrobial stewardship, selective breeding, improved feed and reproductive efficiencies will require a coordinated effort. A validation across ruminant species and production systems, environmental gradients, food matrices, and divergent human health domains is critical. Ultimately, our ability to integrate longitudinal multi-omics data collected under controlled mechanistic experimental structures with epidemiological evidence will be important for reproducibility and enhance causal translational potentials of attributable microbial and metabolic markers.
